# Recent advances in improving the effectiveness and reducing the complications of abortion

**DOI:** 10.12688/f1000research.15441.1

**Published:** 2018-12-02

**Authors:** Sharon Cameron

**Affiliations:** 1 Chalmers Centre, NHS Lothian & University of Edinburgh, 2a Chalmers Street, Edinburgh, EH3 9ES, UK

**Keywords:** abortion, unsafe abortion, global abortion, complications, mifepristone, termination, medical abortion, misoprostol, surgical abortion

## Abstract

When conducted in a legal setting and under safe conditions, abortion is an extremely effective and safe procedure. Tragically, almost half of all abortions that take place in the world are conducted under unsafe conditions, mostly in countries where abortion is illegal or highly restricted. These unsafe abortions are a major cause of maternal death and disability. Restricting a woman’s access to abortion does not prevent abortion but simply leads to more unsafe abortions. Barriers to safe abortion are many but include legal barriers, health policy barriers, shortages of trained healthcare workers, and stigma surrounding abortion. This commentary will consider some recent advances to improve access to safe abortion as well as refinements in abortion methods and service delivery in settings where safe abortion is available that further improve the care and wellbeing of women who seek abortion.

## Introduction

When conducted in a legal setting and under safe conditions, abortion is an extremely safe procedure associated with few complications. US data suggest that it is as safe as or safer than most common outpatient medical procedures and safer than childbirth ^1, 2^. Large studies typically report low rates of major complications (e.g. haemorrhage requiring transfusion, sepsis, and uterine perforation) of less than 1% ^3, 4^. The technology and training required to provide safe abortion is quite straightforward, and both the procedure and the management of minor complications can be safely performed in an outpatient facility rather than a hospital. Evidence-based guidelines from the World Health Organization (WHO) indicate that in most cases abortion can be conducted by a wide range of healthcare providers of primary care level ^5^.

Yet almost half of the 55.7 million abortions that are estimated to take place each year in the world (an estimated 25.1 million abortions) are considered unsafe (17.1 million less safe plus 8.0 million least safe) ^6^. The WHO defines unsafe abortion as a procedure for terminating a pregnancy by persons who are not appropriately trained or use a non-recommended method (less safe) or both (least safe) ^6^. Unsafe abortions lead to a high burden of complications, maternal deaths, and costs. Recent estimates for the global distribution of safe, less-safe, and least-safe abortions show that when the legal status of abortion is considered, the proportion of least-safe abortions is greatest in countries with highly restrictive abortion laws, most of which are developing countries ^6^.

Currently, there is ongoing work to develop consensus on how to improve core outcomes of both medical and surgical abortion (such as ongoing pregnancy and incomplete abortion) and complications of abortion (haemorrhage requiring blood transfusion, infection, etc.) ^7^. This commentary will focus on recent advances for improving completeness of abortion procedures and reducing complications with a focus on improving access to safe abortion, recent developments in medical abortion and surgical abortion, and what more needs to be done.

## Improving access to safe abortion

Aside from legal barriers, a major obstacle to accessing safe abortion worldwide is a lack of trained providers ^5^. The WHO has produced guidelines that recommend the cadre of healthcare provider who can safely and effectively undertake abortion or aspects of abortion care ^5^. Involving a wider range of health workers is an important public health strategy to address the shortages of abortion providers and improve access to safe abortion for women.

The availability of medical methods of abortion, which require relatively little training and which may be safely self-managed by women at home (up to 10 weeks’ gestation) with adequate instruction ^8^, has been a major advance in this respect. This has increased women’s access to safe abortion in many parts of the world ^5^.

Indeed, recent data from WHO/Guttmacher suggest that in settings where abortion is highly restricted, clandestine abortions are now safer because fewer occur by dangerous and invasive methods with more taking place through abortifacient drugs ^6, 9^. Although combination medical abortion, i.e. mifepristone followed by misoprostol, is far more effective than misoprostol alone ^10^, mifepristone is unavailable in many highly restrictive settings, and so misoprostol alone is recommended by WHO when mifepristone cannot be obtained ^11^. Misoprostol is more widely accessible, as it is used to manage a range of conditions (e.g. gastric ulcers, miscarriage, and postpartum haemorrhage). It is also less expensive. However, women who take misoprostol without access to advice from a trained provider can also take risks to their health. There is evidence that trained community workers who distribute misoprostol in restrictive settings can play an important role in improving safety ^12^. In addition, in restrictive settings, telemedicine is helping to improve women’s access to medical abortion over the internet via not-for-profit organisations such as Women on Web, Women Help Women, and Safe2choose. These organisations provide an online consultation for women and information on how to take the medications, the risks, and the signs that indicate the need to seek medical assistance ^13^. Women receive the medication by post in a discrete unmarked package some days to weeks later, depending on where they live. Women on Web report that in the first 10 years of their internet service, over 50,000 women accessed medical abortion from them in this way ^14^. Studies generally indicate that this means of providing medical abortion in the first trimester is associated with high rates of effectiveness and low rates of self-reported complications ^13, 15^, although it should be noted that in studies in highly restrictive settings loss to follow up can be significant. Recently, there has also been growing interest in the use of telemedicine to expand access to medical abortion in settings where abortion is legally available but where it may be difficult to access an abortion provider ^16, 17^.

## Recent developments in medical abortion

### Very early medical abortion

Whilst combination medical abortion with mifepristone and misoprostol is highly effective, studies show that efficacy reduces slightly with increasing gestational age ^18^. Minimising delays in women’s access to safe abortion services so that women who request an abortion can undergo one at as early a gestation as possible is an important strategy to maximise efficacy and improve outcomes. In addition, at early gestations, women may have less pain and bleeding with the procedure. A recent advance has been the development of clinical protocols for what is termed a very early medical abortion (VEMA), i.e. medical abortion at gestations of less than 6 weeks (i.e. too early for a definite intrauterine pregnancy to be visible on ultrasound ^19, 20^). Completeness of VEMA procedures is presumed from a significant fall in serum human chorionic gonadotrophin (HCG) from baseline to treatment ^20^. Data from a large cohort study of over 2,000 women from Sweden and Austria show that the rate of incomplete abortion with VEMA was significantly lower in women having VEMA procedures than among those having a medical abortion at 6–9 weeks (1.8% versus 4.3%, respectively) ^20^. In addition, since VEMA protocols include a baseline and repeat serum HCG ^20^, women who have a higher-than-expected baseline HCG or who do not exhibit the characteristic fall of HCG associated with a successful medical abortion (e.g. a decrease of HCG of >50% after 1 week) are flagged as having a possible ectopic pregnancy ^20^. Although studies show that ectopic pregnancy is extremely uncommon amongst women presenting for medical abortion ^19, 21^ (particularly in the absence of clinical signs or symptoms or risk factors for ectopic pregnancy), this active VEMA management may potentially detect an asymptomatic ectopic pregnancy at an early stage and result in early appropriate intervention that can save lives.

### Second trimester medical abortion

Although surgical abortion in the second trimester (after 12 weeks) using dilation and evacuation (D&E) is a recommended and extremely safe procedure, there is a paucity of providers in many parts of the world who are trained in it. Whilst the efficacy and safety of D&E are dependent on the surgical skill of the provider, the efficacy and safety of medical abortion are dependent on the medication, with relatively minimal training in dosing and route of administration in the second trimester. As a result, a range of providers including non-specialist doctors, midwives, or nurses (with adequate training and back-up) can provide second trimester medical abortion care, thus expanding the availability of safe abortion for women at this stage of gestation ^5^. In some European countries such as Sweden and Scotland, second trimester abortions are almost exclusively medical abortions using mifepristone and misoprostol ^22, 23^.

The combination protocol (mifepristone and misoprostol) in the second trimester has significantly higher efficacy and shorter induction to abortion intervals than misoprostol-only protocols at this stage of pregnancy ^11, 24^. The median induction to abortion interval with the recommended combination regimen is around 6 hours, and 97% will abort within 24 hours ^24^. Surgical intervention rates for incomplete abortion with this regimen are reported to be as low as 5% ^25^. In comparison, for misoprostol-only regimens, the induction to abortion interval in the second trimester is much longer (a median of 10–15 hours), and only 80–90% of women typically abort within 24 hours ^26^.

## Recent developments in surgical abortion

### Cervical priming for first trimester abortion

Surgical abortion using manual or electric vacuum aspiration in the first trimester is not a complex procedure, and the WHO recommends that a range of healthcare providers including an appropriately trained midwife or nurse conduct first trimester aspiration abortions ^5^. Sharp curettage is an outdated method of abortion, and the WHO recommends that surgical abortion in the first trimester should use a plastic aspirator––either a manual handheld syringe or an electrical vacuum aspirator. Existing guidelines have long recommended the routine use of pharmacological and/or mechanical agents to dilate the cervix in procedures that occur in the second trimester, since an open, soft cervix facilitates the passage of a suction aspirator ^11^. Recently, a large multi-country randomised controlled trial (RCT) conducted by the WHO of over 4,800 women undergoing surgical vacuum aspiration in the first trimester showed that pre-treatment with misoprostol can make this earlier gestation procedure even safer ^27^. In this RCT, women having a vacuum aspiration were randomised to either 400 mcg misoprostol vaginally or placebo, 3 hours preoperatively. Misoprostol was associated with a reduction in the risk of both a complication (relative risk [RR] 0.7; 95% confidence interval [Cl] 0.5–0.96) and incomplete abortion needing re-evacuation (RR 0.3; 95% CI 0.2–0.96).

A subsequent RCT has shown that misoprostol administered just 1 hour preoperatively by the sublingual route has an effect on the cervix that is equivalent to that seen when treatment is administered vaginally 3 hours preoperatively ^28^. In addition, administering misoprostol closer to the timing of intervention may be associated with a lower likelihood of some preoperative bleeding and cramping. Shorter preoperative intervals may also allow greater flexibility to schedule procedures.

Numerous studies have reported that a history of previous induced abortion is associated with a subsequent increased risk of preterm delivery, a risk that increases with the number of previous abortions ^29^. It is possible that mechanical dilation of the cervix could lead to cervical injury that could be linked to spontaneous preterm delivery. The routine use of misoprostol prior to surgical abortion (at all gestations) has been commonplace in several parts of Europe such as Scotland, Sweden, and Finland for some years now. Recent registry studies from these countries that have examined reproductive outcomes following abortion have reported that the formerly observed association between previous abortion and preterm labour no longer exists ^30, 31^. It is plausible that widespread use of misoprostol for cervical treatment before all surgical abortion and modern medical methods of inducing abortion may be contributing to this finding.

### Antibiotic prophylaxis

A meta-analysis of RCTs has shown that perioperative antibiotic prophylaxis with surgical abortion is an effective strategy for reducing the risk of post-abortal infection (reduction of 50%) ^32^. Recent studies indicate that post-abortal infection rates are generally less than 1% ^4^. The optimal antibiotic regimen still remains unclear, but commonly used regimens are those that are effective against pathogens such as *Chlamydia trachomatis* and anaerobes. There is evidence that with the use of doxycycline shorter (3-day) courses are as effective as longer (7-day) courses ^33^.

## What more needs to be done?

Countries with restrictive abortion laws have higher unsafe abortion rates than those with liberal laws ^6^. The liberalisation of abortion does not lead to more abortions but makes abortion safer, lowering the costs of treating complications and saving women’s lives ^6^. The legalisation of abortion in countries such as Romania, Nepal, and South Africa was followed by dramatic declines in maternal mortality and morbidity associated with unsafe abortion ^34, 35^.

Abortion stigma, and associated secrecy and shame that surround abortion, is a major factor behind unsafe abortion in many parts of the world ^5, 36^. Stigma prevents women from seeking help when they suffer a complication after an illegal abortion, and this decision to forgo care may lead to death or severe disability. Even in settings where abortion is legal, stigma may result in women delaying an abortion. Such delays push abortions into more advanced gestations, sometimes beyond the legal gestational limit. Stigma also affects providers, who often suffer low morale, lessened prestige, and even ostracism for providing this essential service. Moreover, strong criticism may deter qualified healthcare workers and those in training from undertaking abortion care ^5^. Strategies to address and lessen abortion stigma within our societies and the healthcare professions are therefore necessary.

Given that the vast majority of induced abortions occur because of an unintended pregnancy, it is essential to ensure good access to effective methods of contraception. Making modern methods both affordable and readily available is essential, as is contraceptive information as part of sexuality and relationship education for all. Emergency contraception needs to be available for when regular contraception fails or is not used. Recent data indicate that whilst the numbers of women of reproductive age in developing countries who have an unmet need for modern contraception (i.e. they wish to avoid pregnancy but are not using a modern contraceptive method) have declined in recent years, they remain high at 214 million women ^9^. These women account for 84% of all unintended pregnancies in developing regions.

Fertility can resume quickly after abortion. WHO guidelines recommend that contraceptive counselling and method provision be integrated into comprehensive abortion care ^5, 11^. This includes the most-effective long-acting reversible methods of contraception (LARC) such as the implant and intrauterine contraception, which can be safely initiated immediately after an abortion ^11^. For women having a combination medical abortion, the implant can be inserted at the time of mifepristone administration, and intrauterine contraception can be inserted as soon as expulsion of the pregnancy occurs ^37– 39^. In addition, for women who want to use a LARC method, receiving one at the time of the abortion is associated with higher uptake and fewer subsequent unintended pregnancies than getting one at a later visit ^37, 40, 41^.

## Conclusion

Abortion is an extremely safe procedure and, when it is conducted under safe conditions, complications are uncommon. Liberalising abortion laws around the world is essential for safe abortion but needs to be accompanied by health policies that support implementation of the law, increase the availability of trained providers, and work to remove the stigma of abortion in our societies. Finally, improving the access, availability, and affordability of effective methods of contraception will prevent more unintended pregnancies and thus the abortions, and associated complications, that follow.
